# Efficacy of Minocycline for the Treatment of *Mycoplasma genitalium*

**DOI:** 10.1093/ofid/ofad427

**Published:** 2023-08-11

**Authors:** Emily J Clarke, Lenka A Vodstrcil, Erica L Plummer, Ivette Aguirre, Ranjit S Samra, Christopher K Fairley, Eric P F Chow, Catriona S Bradshaw

**Affiliations:** Melbourne Sexual Health Centre, Alfred Health, Carlton, Victoria, Australia; Melbourne Sexual Health Centre, Alfred Health, Carlton, Victoria, Australia; Central Clinical School, Monash University, Melbourne, Victoria, Australia; Centre for Epidemiology and Biostatistics, Melbourne School of Population and Global Health, University of Melbourne, Melbourne, Victoria, Australia; Melbourne Sexual Health Centre, Alfred Health, Carlton, Victoria, Australia; Central Clinical School, Monash University, Melbourne, Victoria, Australia; Melbourne Sexual Health Centre, Alfred Health, Carlton, Victoria, Australia; Melbourne Sexual Health Centre, Alfred Health, Carlton, Victoria, Australia; Department of Infectious Diseases, Alfred Hospital, Alfred Health, Melbourne, Victoria, Australia; Melbourne Sexual Health Centre, Alfred Health, Carlton, Victoria, Australia; Central Clinical School, Monash University, Melbourne, Victoria, Australia; Melbourne Sexual Health Centre, Alfred Health, Carlton, Victoria, Australia; Central Clinical School, Monash University, Melbourne, Victoria, Australia; Centre for Epidemiology and Biostatistics, Melbourne School of Population and Global Health, University of Melbourne, Melbourne, Victoria, Australia; Melbourne Sexual Health Centre, Alfred Health, Carlton, Victoria, Australia; Central Clinical School, Monash University, Melbourne, Victoria, Australia; Centre for Epidemiology and Biostatistics, Melbourne School of Population and Global Health, University of Melbourne, Melbourne, Victoria, Australia

**Keywords:** antibacterial agents, minocycline, moxifloxacin, *Mycoplasma genitalium*, sexually transmitted infections

## Abstract

**Background:**

High levels of macrolide resistance and increasing fluoroquinolone resistance are making *Mycoplasma genitalium* increasingly difficult to treat. Minocycline is an alternative treatment for patients with macrolide-resistant *M genitalium* infections that have failed moxifloxacin, or for those with fluoroquinolone contraindications or resistance. Published efficacy data for minocycline for *M genitalium* are limited.

**Methods:**

We evaluated minocycline 100 mg twice daily for 14 days at Melbourne Sexual Health Centre (MSHC). Microbial cure was defined as a negative test of cure within 14–90 days after completing minocycline. The proportion cured and 95% confidence intervals (CIs) were calculated, and logistic regression was used to explore factors associated with treatment failure. We pooled data from the current study with a prior adjacent case series of patients with *M genitalium* who had received minocycline 100 mg twice daily for 14 days at MSHC.

**Results:**

Minocycline cured 60 of 90 (67% [95% CI, 56%–76%]) infections. Adherence was high (96%) and side effects were mild and self-limiting. No demographic or clinical characteristics were associated with minocycline failure in regression analyses. In the pooled analyses of 123 patients, 83 (68% [95% CI, 58%–76%]) were cured following minocycline.

**Conclusions:**

Minocycline cured 68% of macrolide-resistant *M genitalium* infections. These data provide tighter precision around the efficacy of minocycline for macrolide-resistant *M genitalium* and show that it is a well-tolerated regimen. With high levels of macrolide resistance, increasing fluoroquinolone resistance, and the high cost of moxifloxacin, access to nonquinolone options such as minocycline is increasingly important for the clinical management of *M genitalium*.

## Teaser

Minocycline is well tolerated, inexpensive, and widely accessible and has 67% efficacy for the treatment of macrolide-resistant *Mycoplasma genitalium*.

*Mycoplasma genitalium* is a sexually transmissible pathogen and is an established cause of nongonococcal urethritis in men [1, 2] and cervicitis, pelvic inflammatory disease, and adverse obstetric outcomes in women [3]. *Mycoplasma genitalium* is becoming increasingly difficult to treat. Macrolide resistance exceeds 50% in some regions [4] and has been shown to exceed 80% among men who have sex with men (MSM) attending Australia's largest sexual health service, Melbourne Sexual Health Centre (MSHC) [5]. At MSHC, moxifloxacin is used as first-line treatment for macrolide-resistant *M genitalium* [6]. If this regimen fails or moxifloxacin is contraindicated, clinicians select from minocycline, pristinamycin, or sitafloxacin, depending on tolerability and contraindications [7–9]. In many settings, access to these options is limited and the cost for some of these drugs is prohibitive. Fluoroquinolone resistance is also on the rise and has reached 25% (ie, for *parC* S83I) in urban Melbourne, further impacting drug selection and cure [10]. As such, nonfluoroquinolone alternatives are being sought.

Minocycline is an option for patients with macrolide-resistant *M genitalium* infections that have failed treatment with fluoroquinolones or for those situations in which fluoroquinolones are contraindicated or not locally accessible. It is widely available, inexpensive, and off-patent. Existing data around the efficacy of minocycline for *M genitalium* are limited to case reports and our previous small series of 35 patients [8]. Our study seeks to provide contemporary estimates around microbial cure, incidence of adverse effects, and adherence to the required 14-day regimen to inform clinician use of this therapeutic option. To provide tighter precision around microbial cure, we pooled our data with a previously reported small case series from our group that immediately preceded this study [8].

## METHODS

We undertook a retrospective analysis of all patients who were prescribed 14 days of minocycline for the treatment of *M genitalium* infection at MSHC between 1 February 2020 and 17 May 2022. Ethics approval was provided by the Alfred Hospital Ethics Committee (approval number 232/16).

Patients were eligible for inclusion in the analysis if they had *M genitalium* detected on a urogenital or anal sample prior to commencing treatment, were treated with minocycline 100 mg twice daily for 14 days for the first time for this infection, completed >50% of the prescribed treatment regimen, completed a test of cure 14–90 days after completion of treatment, and were not deemed to be at high risk for reinfection. In keeping with our previous studies, risk of reinfection was classified as high risk if cases reported condomless penetrative vaginal and/or anal intercourse with an untested and/or untreated or partially treated ongoing partner [6, 11, 12]. Only the first *M genitalium* infection treated with minocycline for each individual during the study period was included in the study, and any subsequent infections were excluded.

It is routine practice at MSHC to be reviewed for an *M genitalium* test of cure 14–28 days after completing any treatment regimen. At the test of cure consultation, clinicians complete a standardized electronic template that captures information regarding resolution of symptoms, adherence, adverse events, sexual activity during and following treatment, and testing and treatment status of sex partner(s).

During the study period, there was a change in the assay used for the diagnosis of *M genitalium* at MSHC. From 1 February 2020 to 28 February 2021, all samples were tested for *M genitalium* and *M genitalium* macrolide resistance using the ResistancePlus MG assay (SpeeDx Pty Ltd, Sydney, Australia), and from 1 March 2021 to 17 May 2022, all samples were tested for *M genitalium* using the *M genitalium* transcription-mediated amplification (TMA) assay (Aptima Hologic Gen-Probe Panther System; Hologic, San Diego, California), which is somewhat more sensitive, detecting lower-load infection [13]. Samples that tested positive for *M genitalium* by TMA were then tested using the ResistancePlus MG assay, so that a macrolide resistance profile was available. MSHC routinely generates a macrolide resistance result for *M genitalium* infections at the time of diagnosis and uses resistance-guided therapy for the treatment of *M genitalium* infections [6, 12]. Microbial cure was defined as a negative test of cure within 14–90 days of completing treatment. The proportion with microbial cure and 95% confidence intervals (CIs) were calculated by exact methods. Logistic regression was used to explore characteristics associated with treatment failure.

To provide greater precision around microbial cure, we then pooled data from the current study with a prior case series of 35 patients with *M genitalium* infection, who had been treated with minocycline 100 mg twice daily for 14 days using the same inclusion criteria as above. This series immediately preceded the current study (May 2018 to February 2020) and had the same eligibility criteria [8].

## RESULTS

**Figure 1.**
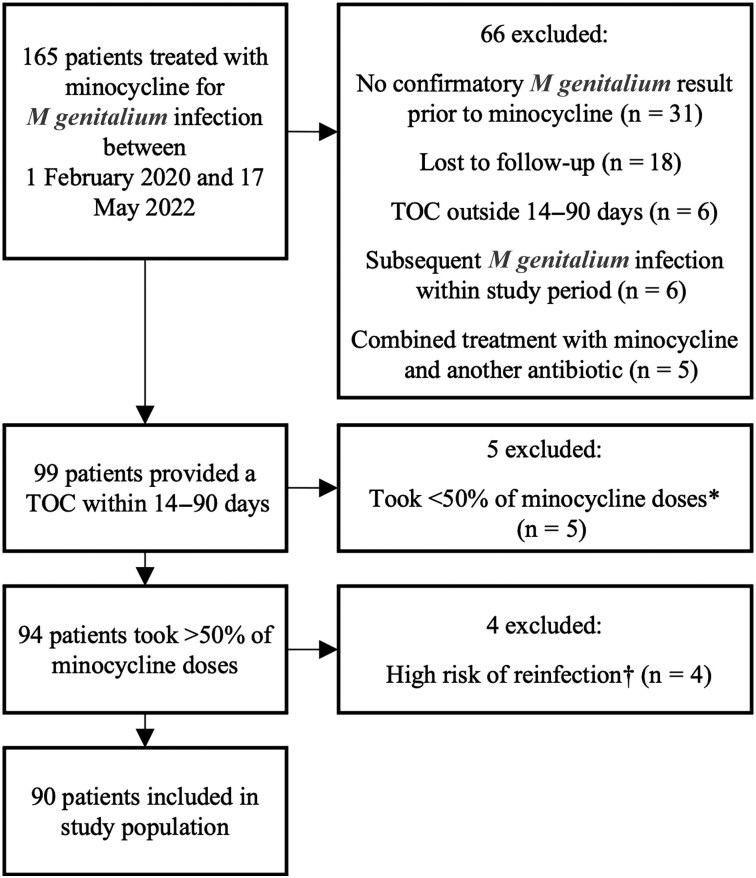
Study selection. *Two patients stopped medication due to side effects (headaches and blurred vision). †Reported condomless penetrative intercourse with an untreated/partially treated ongoing partner. Abbreviation: TOC, test of cure.

From 1 February 2020 to 17 May 2022, 165 patients with macrolide-resistant *M genitalium* were treated with minocycline for 14 days. Patients were excluded from the analysis for the following reasons: no confirmatory test for *M genitalium* undertaken at MSHC prior to receiving minocycline (n = 31), did not return for a test of cure (ie, lost to follow-up, n = 18), did not have a test of cure within the period 14–90 days from treatment completion (n = 6), treatment with minocycline was administered in combination with another antibiotic (n = 5), at high risk of reinfection (n = 4), or took <50% of the 14-day minocycline regimen (n = 5) (Figure 1). In addition, 6 patients received minocycline twice during the study period and their second treatment event was excluded. As a result, 90 patients were included in the final analysis (Figure 1).

### Population Characteristics

The study population had a median age of 29 years (range, 19–53 years) (Table 1). Of the 90 patients included in the study, 25 (27%) were women, 34 (38%) were heterosexual men, and 31 (34%) were MSM. All patients had macrolide-resistant *M genitalium* infections. Two women had multisite infections (cervicovaginal and anal); therefore, the total number of *M genitalium* infections included in the study was 92. A total of 58 (63%) infections were male urethral (diagnosed with first-pass urine samples), 25 (27%) were cervicovaginal (diagnosed with 21 cervicovaginal swabs and 4 first-pass urine samples), and 9 (10%) were anorectal.

**Table 1.** Characteristics of the Study Population

Characteristic	No. (%)
Age, y, median (range)	29 (19–53)
Gender/sexual orientation (n = 90)	
Female^a^	25 (27.2)
Heterosexual male	34 (37.8)
MSM^b^	31 (34.4)
Site of infection (n = 92)	
Cervicovaginal^c^	25 (27.2)
Anorectal	9 (9.8)
Urethral (all male FPU)	58 (63)
Indication for minocycline (n = 90)	
Persistent *Mycoplasma genitalium*, failed prior treatment	
Asymptomatic	18 (20)
Male urethritis	36 (40)
Rectal symptoms^d^	2 (2.2)
Female genital/pelvic symptoms^e^	9 (10)
First treatment for *M genitalium*	
Male urethritis	18 (20)
Rectal symptoms^f^	2 (2.2)
Female genital/pelvic symptoms^g^	1 (1.1)
Asymptomatic	4 (4.4)
Prior antibiotic treatment (n = 90)	
No prior treatment^h^	25 (27.8)
Prior treatment^i^	65 (72.2)
1 prior treatment regimen	35 (38.9)
≥2 prior treatment regimens	30 (33.3)
Adherence	
No missed doses documented	86 (95.6)
Missed 1–6 doses	4 (4.4)
Side effects	
Dizziness/lightheadedness	8 (8.9)
Nausea	5 (5.6)
Headache	5 (5.6)
Fatigue/lethargy	4 (4.4)
Derealization/“brain fog”/low mood	4 (4.4)
Abdominal pain	3 (3.3)
Diarrhea	2 (2.2)
Reflux/heartburn	2 (2.2)
Other^j^	7 (7.8)

Abbreviations: FPU, first-pass urine; MSM, men who have sex with men.

a Two patients had multisite infections.

b Includes 1 man having sex with a transgender woman.

c Represents 21 cervicovaginal swabs and 4 FPU samples.

d Mild dyschezia (n = 1), discharge (n = 1).

e Urinary symptoms (n = 1), abnormal bleeding (n = 3), abnormal bleeding + discharge (n = 1), pelvic pain + abnormal bleeding + altered vaginal discharge (n = 1), pelvic pain (n = 2), altered vaginal discharge (n = 1).

f Itch (n = 1), mild discomfort (n = 1).

g Pelvic pain (n = 1).

h Sixteen of 25 patients had received doxycycline prior to commencing minocycline (median, 7 days). Fourteen were symptomatic and 2 were contacts of infection.

i Six of 65 patients had received doxycycline prior to commencing minocycline (median, 7 days). Sixty-four of these patients had previously received and failed treatment with moxifloxacin. Additional antibiotics that patients had been exposed to included azithromycin (n = 25), sitafloxacin (n = 12), pristinamycin (n = 9), and lefamulin (n = 2).

j Includes rash, bloating, sleep disturbance, dry eyes, and sun sensitivity.

For 25 (28%) patients, minocycline was their first treatment for macrolide-resistant *M genitalium*. The most common reasons that minocycline was used for first-line treatment were drug–drug interactions with moxifloxacin, previous adverse reactions to quinolones, or other medical contraindications. Of these, 18 (20%) were men who presented with urethritis, 4 patients were asymptomatic contacts, 2 men presented with mild rectal symptoms, and 1 woman presented with pelvic pain. The remaining 65 (72%) patients were treated with minocycline for a persistent *M genitalium* infection that had failed other antimicrobials: 36 (40%) men presented with urethritis, 18 (20%) men were asymptomatic, 2 men had rectal symptoms, and 9 (10%) were women with genital/pelvic symptoms. Of these 9 women, 3 presented with abnormal vaginal bleeding, 2 with pelvic pain, 1 with altered vaginal discharge, 1 with urinary symptoms, 1 with abnormal vaginal discharge and bleeding, and 1 with pelvic pain, abnormal bleeding, and vaginal discharge.

Of the 65 (72%) patients who had failed a prior antibiotic regimen(s), 35 (39%) had received 1 prior regimen, and 30 (33%) patients had received ≥2 prior regimens. A total of 62 (69%) patients had failed a moxifloxacin-containing regimen. Other prior regimens received contained sitafloxacin, azithromycin, pristinamycin, and lefamulin.

### Microbial Cure

Of the 90 patients included in the study population, 60 (66.7% [95% CI, 53.3%–78.3%]) experienced microbial cure within 14–90 days of completing minocycline. We next examined the impact of epidemiological, clinical, and behavioral factors on microbial cure by logistic regression analysis (Table 2). While small numbers limited our ability to determine impact of gender/sexual orientation on cure, the odds of failing minocycline were similar in all 3 subgroups (*P* > .7): 17 of 25 females (68% [95% CI, 46.5%–85.1%]) experienced microbial cure, along with 22 of 34 heterosexual males (64.7% [95% CI, 46.5%–80.3%]) and 21 of 31 MSM (67.7% [95% CI, 48.6%–83.3%]). When examining microbial cure by anatomical site, 17 of 25 cervicovaginal infections (68.0% [95% CI, 46.5%–85.1%]), 7 of 9 rectal infections (77.8% [95% CI, 40.0%–97.2%]), and 38 of 58 male urethral infections (65.6% [95% CI, 51.9%–77.5%]) were cured. There was no significant difference in odds of minocycline failure between these 3 sites (*P* > .5). There was also no significant difference in the odds of failure between individuals with and without symptoms (63.6% [95% CI, 40.7%–82.8%] and 67.6% [95% CI, 55.2%–78.5%], respectively; odds ratio [OR], 0.84 [95% CI, .31–2.29]; *P* = .729). Of the 25 patients who had not received any prior treatment for *M genitalium*, 18 experienced microbial cure (72% [95% CI, 50.1%–87.9%]) compared to 42 of the 65 previously treated patients (64.6 [95% CI, 51.8%–76.1%]). There was no statistical difference in the odds of failure between these 2 groups (OR, 1.41 [95% CI, .51–3.87]; *P* = .507). Last, of the 86 patients who did not report any missed doses of minocycline, 59 experienced microbial cure (68.6% [95% CI, 57.7%–78.2%]), whereas only 1 of the 4 patients who reported missing 1–6 doses of minocycline experienced microbial cure (25% [95% CI, .6%–80.1%]); there was no significant difference in the odds of failure between these 2 groups (OR, 6.55 [95% CI, .65–65.95]; *P* = .121), but small numbers again affected this comparison.

**Table 2.** Factors Associated With Failure (N = 90)

Factor	Cured	Failed	OR (95% CI)	*P* Value^a^
	No. (% [95% CI])	No. (% [95% CI])		
Total patients	60 (66.7 [53.3–78.3])	30 (33.3 [17.3–52.8])	…	
Gender/sexual orientation				
Female	17 (28.3 [17.5–41.4])	8 (26.7 [12.3–45.9])	Ref	
Heterosexual male	22 (36.7 [24.6–50.1])	12 (40 [22.7–59.4])	1.16 (.39–3.47)	.792
MSM^b^	21 (35 [23.1–48.4])	10 (33.3 [17.3–52.8])	1.01 (.33–3.13)	.984
Site of infection^c^				
Cervicovaginal	17 (27.4 [16.9–40.2])	8 (26.7 [12.3–45.9])	Ref	
Anorectal	7 (11.3 [4.7–21.9])	2 (6.7 [.8–22.1])	0.71 (.11–4.47)	.719
Urethral (male FPU)	38 (61.3 [48.1–73.4])	20 (66.7 [47.2–82.7])	1.38 (.47–4.05)	.563
Presence of symptoms				
Asymptomatic	14 (23.3 [13.4–36.0])	8 (26.7 [12.3–45.9])	Ref	
Symptomatic	46 (76.7 [64.0–86.6])	22 (73.3 [54.1–87.7])	0.84 (.31–2.29)	.729
Previous treatment				
No prior treatment	18 (30 [18.8–43.2])	7 (23.3 [9.9–42.3])	Ref	
Any prior treatment	42 (70 [56.8–81.2])	23 (76.7 [57.7–90.1])	1.41 (.51–3.87)	.507
Adherence				
No reported missed doses	59 (98.3 [91.1–100])	27 (90 [73.5–97.9])	Ref	
Missed 1–6 doses	1 (1.7 [.4–8.9])	3 (10 [2.1–26.5])	6.55 (.65–65.95)	.121

Abbreviations: CI, confidence interval; FPU, first-pass urine; MSM, men who have sex with men; OR, odds ratio.

a *P* values calculated using logistic regression. For site of infection, the analysis was clustered by participant.

b Includes 1 man having sex with a transgender woman.

c For site of infection, the sample size is 92.

### Adherence to Treatment and Adverse Effects

Adherence and adverse effects were reported in the final study population where >50% adherence to minocycline was required. Among the final study population, 86 patients (96%) reported taking all minocycline doses. Side effects were mostly mild and self-limiting and predominantly affected the gastrointestinal or central nervous systems. A total of 8 patients reported dizziness, 5 patients reported nausea, 5 reported headache, 4 reported fatigue/lethargy, and 4 reported a feeling of derealization, “brain fog,” or low mood (Table 1). Interestingly, of the 165 prescriptions of minocycline provided by MSHC between 1 February 2020 and 17 May 2022 before assessment for eligibility for this study (Figure 1), only 5 (3%) people reported <50% adherence and were excluded from this study, and 2 of these 5 patients had stopped the medication prematurely due to side effects (reported headache and/or blurred vision, which resolved).

### Pooled Data

With the aim of increasing accuracy around efficacy estimates for 14 days of minocycline, we pooled data with our prior case series of 35 patients who had been treated with the same regimen at MSHC in the period that immediately preceded this study (ie, May 2018–February 2020) [8]. Of the 35 patients in that series, 33 fulfilled the eligibility criteria for the current study, creating a pooled dataset of 123 patients with macrolide-resistant *M genitalium* who were treated with minocycline (Table 3). Of the 123 patients, overall cure was 67.5% (95% CI, 58.4%–75.6%). We undertook logistic regression analyses in this larger dataset to determine if any factors were associated with microbial failure, but no statistically significant associations between patient characteristics and treatment failure were found (*P* > .169).

**Table 3.** Factors Associated With Failure When Pooled With a Prior Population

Factor	Pooled Data (n = 123)			
	Cured	Failed	OR (95% CI)	*P* Value^a^
	No. (% [95% CI])	No. (% [95% CI])		
Total patients	83 (67.5 [58.4–75.6])	40 (32.5 [24.4–41.6])	…	
Gender/sexual orientation				
Female	27 (32.5 [22.6–43.7])	11 (27.5 [14.6–43.9])	Ref	
Heterosexual male	30 (36.1 [25.9–47.4])	17 (42.5 [27–59.1])	1.39 (.55–3.49)	.482
MSM^b^	26 (31.3 [21.6–42.4])	12 (30 [16.6–46.5])	1.13 (.43–3.02)	.803
Site of infection^c^				
Cervicovaginal	27 (31.8 [22.1–42.8])	11 (27.5 [14.6–43.9])	Ref	
Anorectal	7 (8.2 [3.3–16.2])	2 (5.0 [.6–16.9])	0.70 (.13–3.85)	.683
Urethral (male FPU)	51 (60 [48.8–70.5])	27 (67.5 [50.9–81.4])	1.30 (.56–3.03)	.544
Presence of symptoms				
Asymptomatic	17 (20.5 [12.4–30.8])	9 (22.5 [10.8–38.5])	Ref	
Symptomatic	66 (79.5 [69.2–87.6])	31 (77.5 [61.5–89.2])	0.89 (.36–2.21)	.797
Previous treatment				
No prior treatment	22 (26.5 [17.4–37.3])	8 (20 [9.1–35.6])	Ref	
Any prior treatment	61 (73.5 [62.7–82.6])	32 (80 [64.4–90.9])	1.35 (.54–3.40)	.517
Adherence				
No reported missed doses	80 (96.4 [89.8–99.2])	36 (90 [76.3–97.2])	Ref	
Missed 1–6 doses	3 (3.6 [.8–10.2])	4 (10 [2.8–23.7])	2.96 (.63–13.93)	.169

Abbreviations: CI, confidence interval; FPU, first-pass urine; MSM, men who have sex with men; OR, odds ratio.

a *P* values calculated using logistic regression. For site of infection, the analysis was clustered by participant.

b Includes 1 man having sex with a transgender woman.

c For site of infection, the sample size is 125 for the pooled population.

## DISCUSSION

This retrospective real-world analysis of the effectiveness of minocycline found that 66.7% (95% CI, 53.3%–78.3%) of macrolide-resistant *M genitalium* infections were cured with a 14-day regimen of minocycline. While this case series of 90 patients is the largest reported to date, our findings are consistent with the previous published series of 35 patients, which reported that 71% (95% CI, 54%–85%) were cured following minocycline [8]. This finding of 67% cure following minocycline is slightly lower than the cure reported following pristinamycin (75% [95% CI, 66%–82%]) [8, 9] and, importantly, slightly higher than cure following moxifloxacin (59% [95% CI, 46.7%–69.9%]) in the presence of S83I *parC* mutations [10].

In the era of rising macrolide and fluoroquinolone resistance, clinicians increasingly need to access alternatives to fluoroquinolones. Having more confidence and precision around cure for minocycline enables clinicians to discuss treatment options with patients who have persistent *M genitalium* infections or drug contraindications, as well as in settings with limited drug options and/or high prevalence of fluoroquinolone resistance.

Minocycline is a second-generation, semi-synthetic tetracycline analogue that has been on the market since the late 1960s [14, 15], and there is a long history of data on minocycline safety and tolerability [14, 15]. Minocycline is generally well tolerated. Common side effects tend to be neurological (eg, weakness, dizziness, headache) and gastrointestinal (eg, abdominal cramps) [16]. Product labeling states the incidence of common side effects as 9% for dizziness, 9% for fatigue, 5% for pruritus, and 4% for malaise [17]. There is a known risk of idiopathic intracranial hypertension with tetracyclines, which presents as headache, tinnitus, and transient visual obscurations; however, this is a rare adverse effect. A systematic review of available literature from 1900 through 2019 identified 32 cases of idiopathic intracranial hypertension associated with minocycline use, almost all of which were associated with long-term use for acne treatment [18]. Patients in our analysis reported mostly mild and self-limiting adverse effects and we found that the incidence of reported adverse effects in the study population was similar to available published data outlined above. Reassuringly, of the 165 prescriptions of minocycline provided by MSHC between February 2020 and May 2022, only 2 patients stopped their medication prematurely due to side effects (headaches and blurred vision, which resolved completely and were not deemed to be idiopathic intracranial hypertension).

A considerable advantage of minocycline compared to moxifloxacin, sitafloxacin, and pristinamycin is that it is widely accessible at low cost. A course of minocycline comes at a direct cost of AU$9 (US$6.4) to pharmacies in Australia. In contrast, antibiotics such as moxifloxacin and pristinamycin cost up to AU$87 (US$61) and AU$207 (US$146), respectively. In addition to this, the majority of these alternative regimens are not readily available in community pharmacies. Furthermore, pristinamycin and sitafloxacin both require an application to the Australian Therapeutic Goods Administration, the equivalent of the United States (US) Food and Drug Administration and the United Kingdom Medicines and Healthcare Products Regulatory Agency, limiting access to these drugs to specialized services. In other countries, alternative regimens may not be accessible at all; for example, at the time of writing, pristinamycin is not available in the US and sitafloxacin is not available in the US or Europe. Based on these reasons, minocycline offers genuine opportunities for clinicians in high-, low-, and middle-income countries to treat macrolide-resistant *M genitalium*.

In contrast to macrolides and quinolones, the mechanisms of tetracycline failure in the treatment of *M genitalium* are not well understood. Based on in vitro data, tetracyclines appeared highly promising for the treatment of *M genitalium*, with minimum inhibitory concentrations (MICs) indicating that many isolates of *M genitalium* should be doxycycline susceptible [19, 20]. The MICs of minocycline for reference and clinical strains were slightly lower than those of doxycycline [21], indicating it may be more effective, although structural differences between the 2 antibiotics also likely impact the efficacy of each drug in vivo [21]. While there are no large clinical studies using minocycline for the treatment of *M genitalium*, randomized trials largely conducted over a decade ago consistently found that the tetracycline doxycycline was inferior to azithromycin in eradicating *M genitalium* with proportion cured in the order of 20%–40% [4]. However, no molecular mechanisms of resistance in the 16S ribosomal RNA gene have been found to consistently explain the low efficacy of doxycycline [22], and no correlation between MICs and microbial cure has been demonstrated [20].

Importantly, our analysis did not find any statistically significant associations with minocycline failure, although our sample size is likely to have impacted on these comparisons. It is interesting to note that of the 9 anorectal infections, 7 (77.8%) were cured compared to the 17 of 25 (68.0%) cervicovaginal infections (OR, 0.71 [95% CI, .11–4.47]; *P* = .719). It would be important to establish if there is indeed a difference in efficacy by anatomical site with a larger sample size. It has been shown that minocycline is more lipophilic than tetracycline and demonstrates greater tissue penetration, including into the intestinal epithelium [14, 23, 24], which could support a potential increased efficacy for anorectal infections.

In addition to sample size, this study has other limitations. This was a retrospective analysis that relied upon patients’ self-reported recall of information regarding adherence, sexual activity, and adverse effects of treatment. There is also the possibility of attrition bias, as 18 of the initial population of 165 patients (10.9%) were lost to follow-up. This could result in an overreporting of failures, as those with persistent symptoms might be more likely to attend follow-up, although based on our prior studies this cohort had a very high rate of return for test of cure. As participants comprised of sexually transmitted infection clinic attendees, these findings may not be generalizable to the community.

In conclusion, our study provides clinicians with increased precision and confidence around microbial cure (68%) for minocycline for macrolide-resistant infection and shows that it is a well-tolerated regimen associated with predominantly mild side effects in a minority of patients. In the context of rising quinolone resistance, which has reached 25% (ie, for *parC* S83I) in urban Melbourne [10], the availability of nonquinolone agents such as minocycline is becoming increasingly important. In some regions, particularly in the Asia-Pacific region, we may have already reached a point where the efficacy of minocycline is equal to or superior to moxifloxacin [25, 26]. For low- and middle-income countries, the availability of an inexpensive generic that will cure in the order of 2 of 3 macrolide-resistant infections may be welcomed by clinicians and patients.

## Notes

***Acknowledgments.*** We sincerely thank the staff at Melbourne Sexual Health Centre for collecting the clinical data used in analyses, as well as Michelle Doyle, who conducted the earlier series with which we pooled data. E. J. C. is a fellow of the Royal Australian College of General Practitioners and an advanced trainee with the Australasia Chapter of Sexual Health Medicine.

***Patient consent*.** Ethics approval was obtained from the Alfred Health ethics committee (approval number 232/16). Written patient consent was not required as this was a retrospective audit.

***Financial support.*** C. K. F. and C. S. B. are supported by Australian National Health and Medical Research Council (NHMRC) Leadership Investigator Grants (grant numbers GNT1172900 and GNT1173361, respectively), and E. P. F. C. is supported by an NHMRC Emerging Leadership Investigator Grant (grant number 1172873).
